# Supplementary material for: Artificial intelligence-rationalized balanced PPARα/γ dual agonism resets dysregulated macrophage processes in inflammatory bowel disease
Source: Commun Biol. 2022 Mar 14;5:231. doi: 10.1038/s42003-022-03168-4 (PMC8921270; doi:10.1038/s42003-022-03168-4)
Supplement: Supplementary file 2 — Supplementary Information [file 42003_2022_3168_MOESM2_ESM.pdf]

SUPPLEMENTARY ONLINE MATERIALS

Artificial Intelligence-rationalized balanced PPARα/γ dual agonism resets the dysregulated macrophage processes in inflammatory bowel disease

Authors: Gajanan D. Katkar[1], Ibrahim M. Sayed[2,3], Mahitha Shree Anandachar[2], Vanessa Castillo[1], Eleadah Vidales[1], Daniel Toobian[1], Fatima Usmani[2], Joseph R. Sawires[4], Geoffray Leriche[4], Jerry Yang[4], William J. Sandborn[5]*, Soumita Das[2]*, Debashis Sahoo[6,7,8]* and Pradipta Ghosh[1, 5, 8, 9]*

Affiliations:
Affiliations:
[1]Department of Cellular and Molecular Medicine, University of California San Diego, San Diego, USA.
[2]Department of Pathology, University of California San Diego, San Diego, USA.
[3]Department of Medical Microbiology and Immunology, Faculty of Medicine, Assiut University, Assiut, Egypt.
[4]Department of Chemistry and Biochemistry, University of California San Diego, San Diego, USA.
[5]Department of Medicine, University of California San Diego, San Diego, USA.
[6]Department of Computer Science and Engineering, Jacob's School of Engineering, University of California San Diego, San Diego, USA.
[7]Department of Pediatrics, University of California San Diego, San Diego, USA.
[8]Rebecca and John Moore Comprehensive Cancer Center, University of California San Diego, San Diego, USA.
[9]Veterans Affairs Medical Center, La Jolla, San Diego, USA.

Runnning title: AI-guided macrophage modulation in IBD

*__Correspondence to__:

William J. Sandborn, M.D.; Professor, Department of Medicine, University of California San Diego; 9500 Gilman Drive, MC 0956, La Jolla, CA 92093-0831.
Phone: 858-657-5331, Email: wsandborn@health.ucsd.edu

Soumita Das, Ph.D.; Associate Professor, Department of Pathology, University of California, San Diego; 9500 Gilman Drive, George E. Palade Bldg, Rm 256, 239; La Jolla, CA 92093.
Phone: 858-246-2062 (office): **Email:** sodas@ucsd.edu

Debashis Sahoo, Ph.D; Assistant Professor, Department of Pediatrics, University of California San Diego; 9500 Gilman Drive, MC 0730, Leichtag Building 132; La Jolla, CA 92093-0831.
Phone: 858-246-1803: Fax: 858-246-0019: Email: dsahoo@ucsd.edu

Pradipta Ghosh, M.D.; Professor, Departments of Medicine, and Cell and Molecular Medicine, University of California San Diego; 9500 Gilman Drive (MC 0651), George E. Palade Bldg, Rm 232, 239; La Jolla, CA 92093. Phone: 858-822-7633: Fax: 858-822-7636: Email: prghosh@ucsd.edu

CATALOG OF SUPPLEMENTARY MATERIALS

1. *Supplementary Figures and Legends (S1-S12, Page 3-14)*
2. *Supplementary Tables (1-5, Page 15-21)*
3. *Supplementary References (Page 22-23)*

SUPPLEMENTARY FIGURES

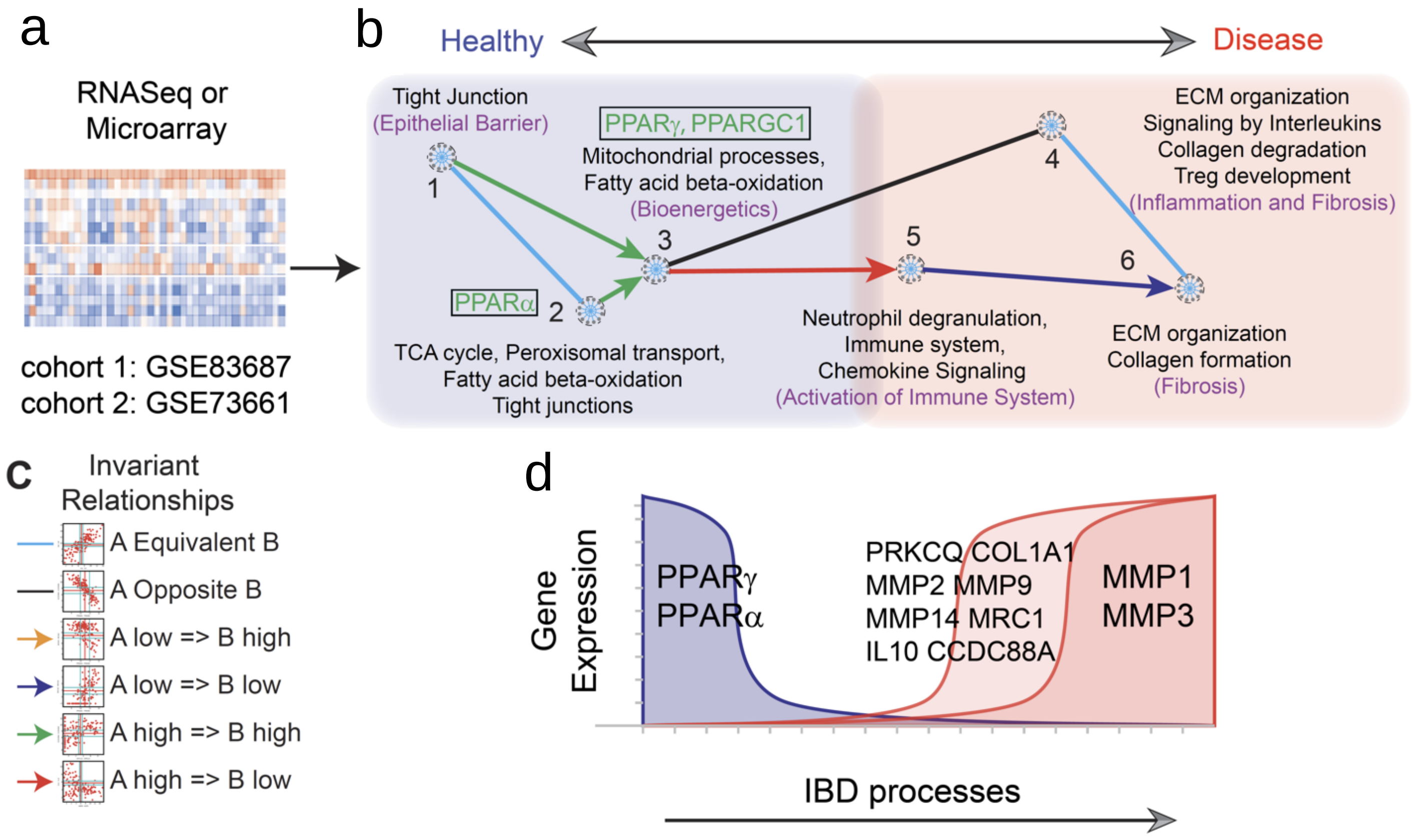

Supplementary Figure 1: Boolean network map of continuum states in Inflammatory Bowel Disease (a.k.a, IBD-map) and the position of PPARA/G targets within the map.
(**a**) Boolean network analysis was performed on IBD datasets (GSE83687 and GSE73661) to identify pathways and gene clusters during IBD progression. (**b**) Genes with similar expression profiles were organized into clusters, and relationships between clusters represented as color-coded edges connecting clusters. Reactome pathway of each cluster of IBD-map was performed to understand pathophysiological cellular processes that are enriched during IBD progression. PPARA is present within cluster #2 and PPARG is present within cluster #3. (**c**) Boolean networks contain six possible Boolean relationships between genes (invariant relationships). (**d**) Schematic showing the gene expression of selected genes within the normal to IBD disease progression.

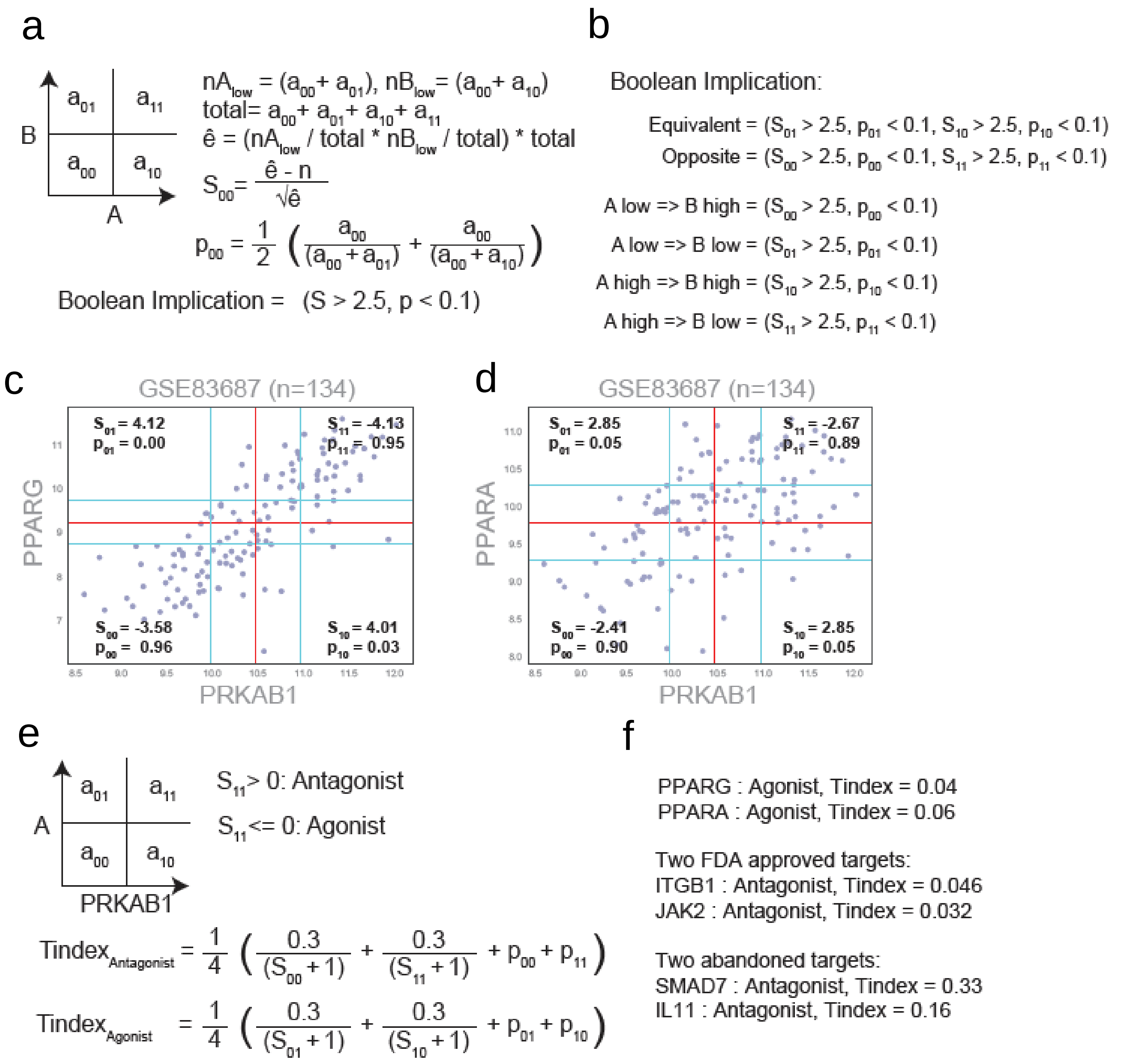

$$nA_{low} = (a_{00} + a_{01}),\ nB_{low} = (a_{00} + a_{10})$$
$$total = a_{00} + a_{01} + a_{10} + a_{11}$$
$$\hat{e} = (nA_{low} / total * nB_{low} / total) * total$$
$$S_{00} = \frac{\hat{e} - n}{\sqrt{\hat{e}}}$$
$$p_{00} = \frac{1}{2}\left(\frac{a_{00}}{(a_{00}+a_{01})} + \frac{a_{00}}{(a_{00}+a_{10})}\right)$$
$$Tindex_{Antagonist} = \frac{1}{4}\left(\frac{0.3}{(S_{00}+1)} + \frac{0.3}{(S_{11}+1)} + p_{00} + p_{11}\right)$$
$$Tindex_{Agonist} = \frac{1}{4}\left(\frac{0.3}{(S_{01}+1)} + \frac{0.3}{(S_{10}+1)} + p_{01} + p_{10}\right)$$

Supplementary Figure 2: Computation of therapeutic index in a target report card. **(a)** BooleanNet statistic. Evaluating Boolean implication relationship between gene A and B. a_{ij} is the number of samples in the respective quadrants. nA/B_{low} is number of samples where A/B is low. S_{00} = BooleanNet statistic and p_{00} = error rate to test sparsity for the bottom left quadrant. $S > 2.5$ and $p < 0.1$ is used to test whether each quadrant is sparse. False Discovery Rate is computed by randomizing the data several times and computing the ratio of an average number of relationships found in randomized data to the original data. **(b)** Deriving Boolean implication relationships using BooleanNet statistic. **(c-d)** Scatterplots between *PRKAB1*, *PPARG* (c) and *PPARA* (d) in GSE83687 (n = 134) with the StepMiner thresholds (red) and noise margin (+/- 0.5, blue) in both X and Y-axes. BooleanNet statistic (S, p) is computed for each quadrant. **(e)** Because *PRKAB1* confers the desirable phenotype when engaged with an Agonist, gene A is considered Antagonist if $S_{11} > 0$ (Top-right quadrant have fewer points than expected) and Agonist otherwise. The therapeutic index (Tindex) is computed separately for Antagonist and Agonist as shown below. **(f)** The therapeutic index (T-index) value of PPARG, PPARA, two FDA approved targets (ITGB1, JAK2), two abandoned targets (SMAD7, IL11) is computed using the formula in panel e.

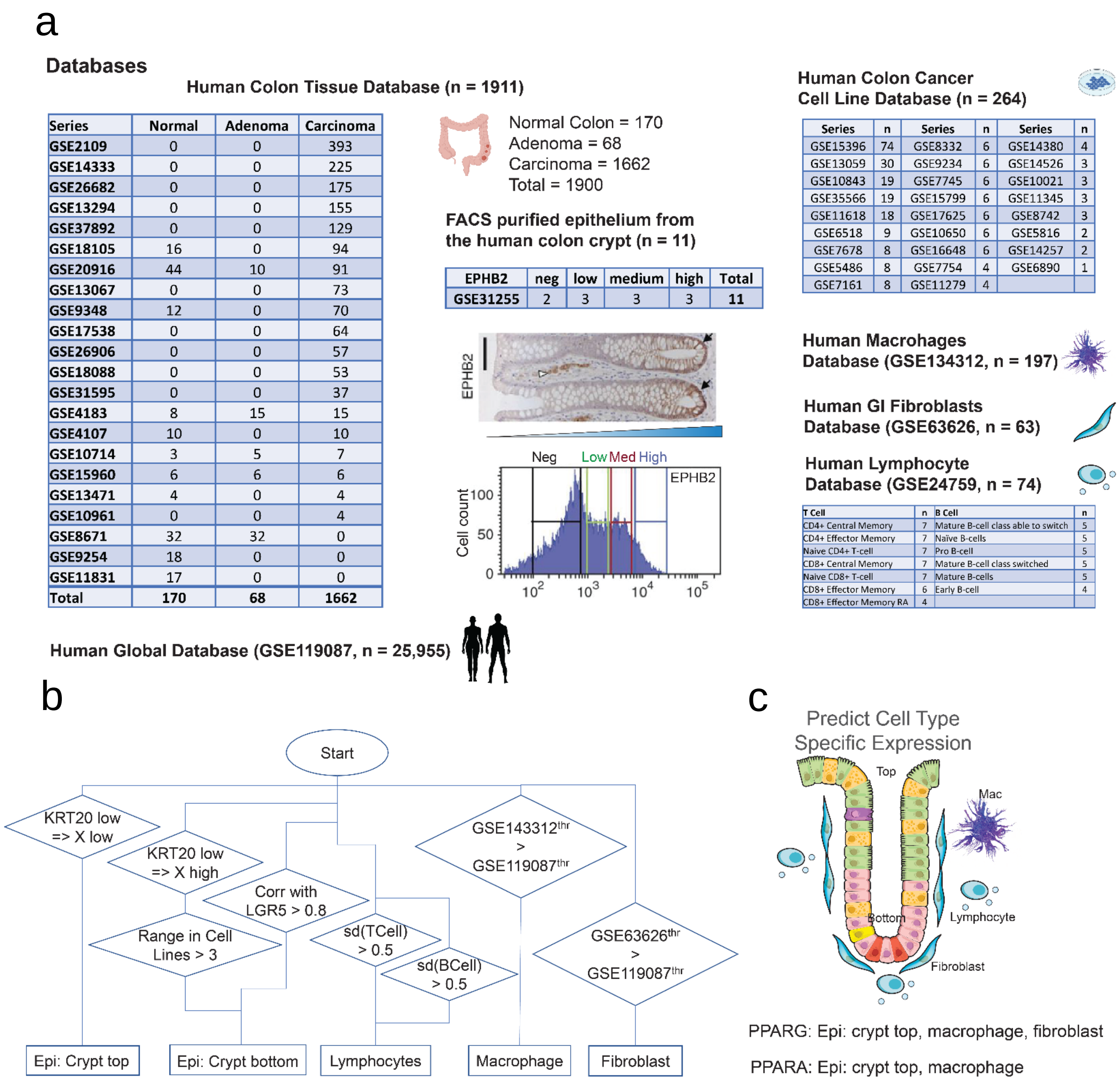

Supplementary Figure 3: Prediction of cell type specific expression patterns. **(a)** Gene expression databases used for predicting cell type specific expression patterns. Human colon tissue database (n = 1911) is derived from the "Human Colon Global Database after purging using EpCAM and Albumin" restricted to Human U133 Plus 2.0 Affymetrix platform as published previously (Dalerba, Sahoo et al. 2016, NEJM, PMID: 26789870) with additional 68 Adenoma samples and purified FACS samples of human colon crypts (GSE31255). A database of human colon cancer cell lines (n = 264) was prepared by pooling 26 independent datasets. Macrophage, and Fibroblast databases were prepared from GSE134312 and GSE63626, respectively. B cell and T cell samples from GSE24759 was used to prepare the human Lymphocyte database. Human Global Database (GSE119087, n = 25,955) is used to identify high and low expression patterns relative to other tissue types for each gene. **(b)** Flow chart for the prediction of cell type specific expression patterns. Boolean implication "*KRT20* low => X low" in the human colon tissue database (n = 1911) is used to identify the top of the crypt specific expression patterns. Boolean implication "*KRT20* low => X high" in the human colon tissue database (n = 1911), dynamic range > 3 in human colon cancer cell lines (n = 264), and correlation with *LGR5* > 0.8 in GSE31255 is used to identify bottom of the crypt specific expression patterns. Standard deviation > 0.5 in B cell and T cell samples from GSE24759 is used to predict if a gene is expressed in lymphocytes. Macrophage and Fibroblast specific expression pattern is predicted by comparing StepMiner thresholds of GSE134312 and GSE63626 with GSE119087. **(c)** Cell type specific expression patterns for *PPARG* and *PPARA* is computed using the flow chart in panel b. Their actions potentially overlap in the epithelium at the crypt top and in macrophages, and hence, any pharmacologic impact of their dual agonism may be appreciated in these cell types.

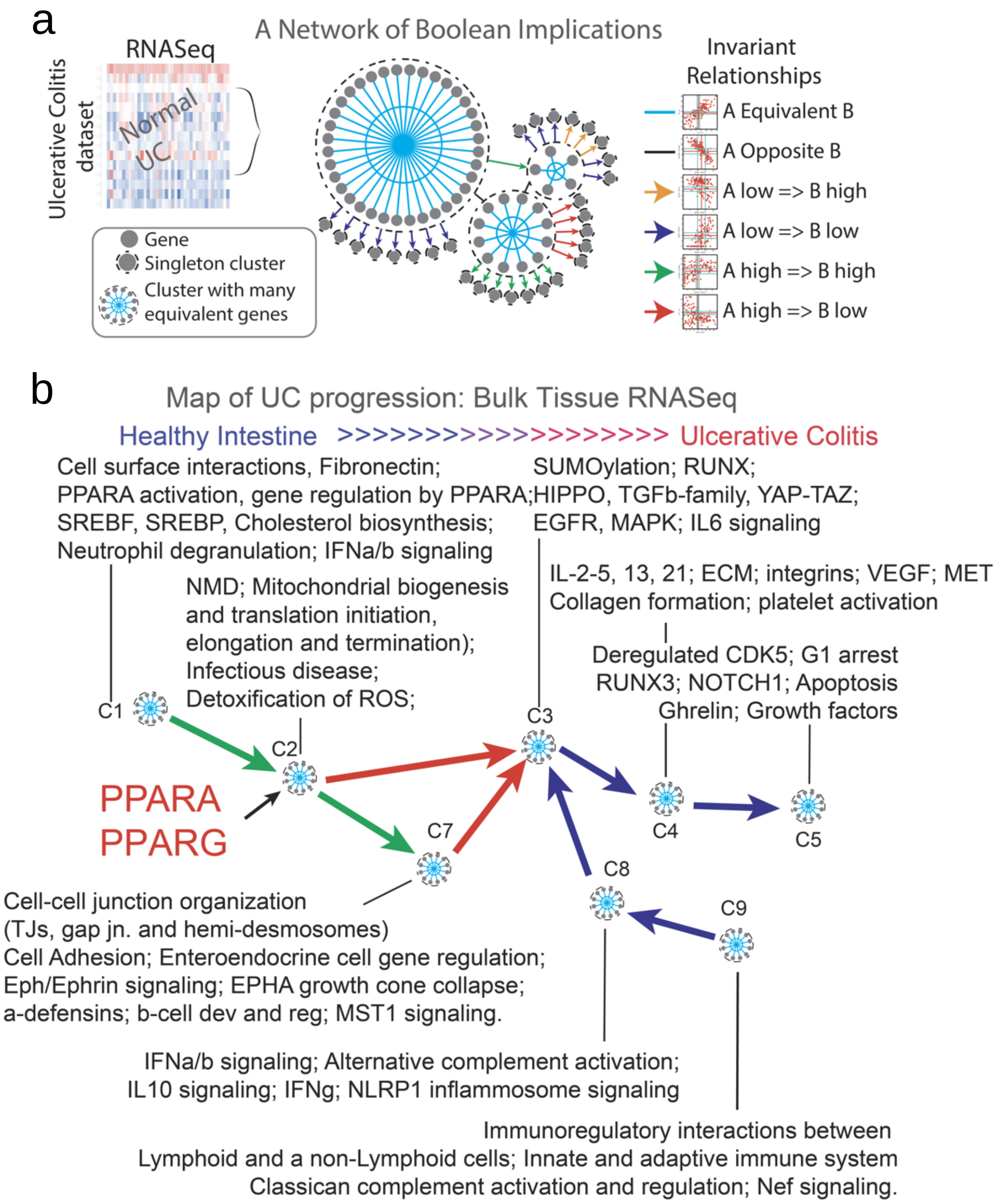

Supplementary Figure 4: Boolean network map of continuum states in Ulcerative Colitis (a.k.a, UC-map) and the position of PPARA/G targets within the map.
(a) Boolean network analysis was performed on Ulcerative colitis datasets to identify pathways and gene clusters during UC progression. Boolean networks contain six possible Boolean relationships between genes (invariant relationships top right) and genes with similar expression profiles were organized into clusters, and relationships between clusters represented as color-coded edges connecting clusters. **(b)** Schematic of reactome pathway of each cluster of UC-map were performed to understand pathophysiological cellular processes that are enriched during UC progression. PPARA and PPARG are both located in cluster #2. Their stimulation with dual agonists is expected to impact both continuum paths, C2→C3→C4→C5 and C2→C7→C3→C4→C5.

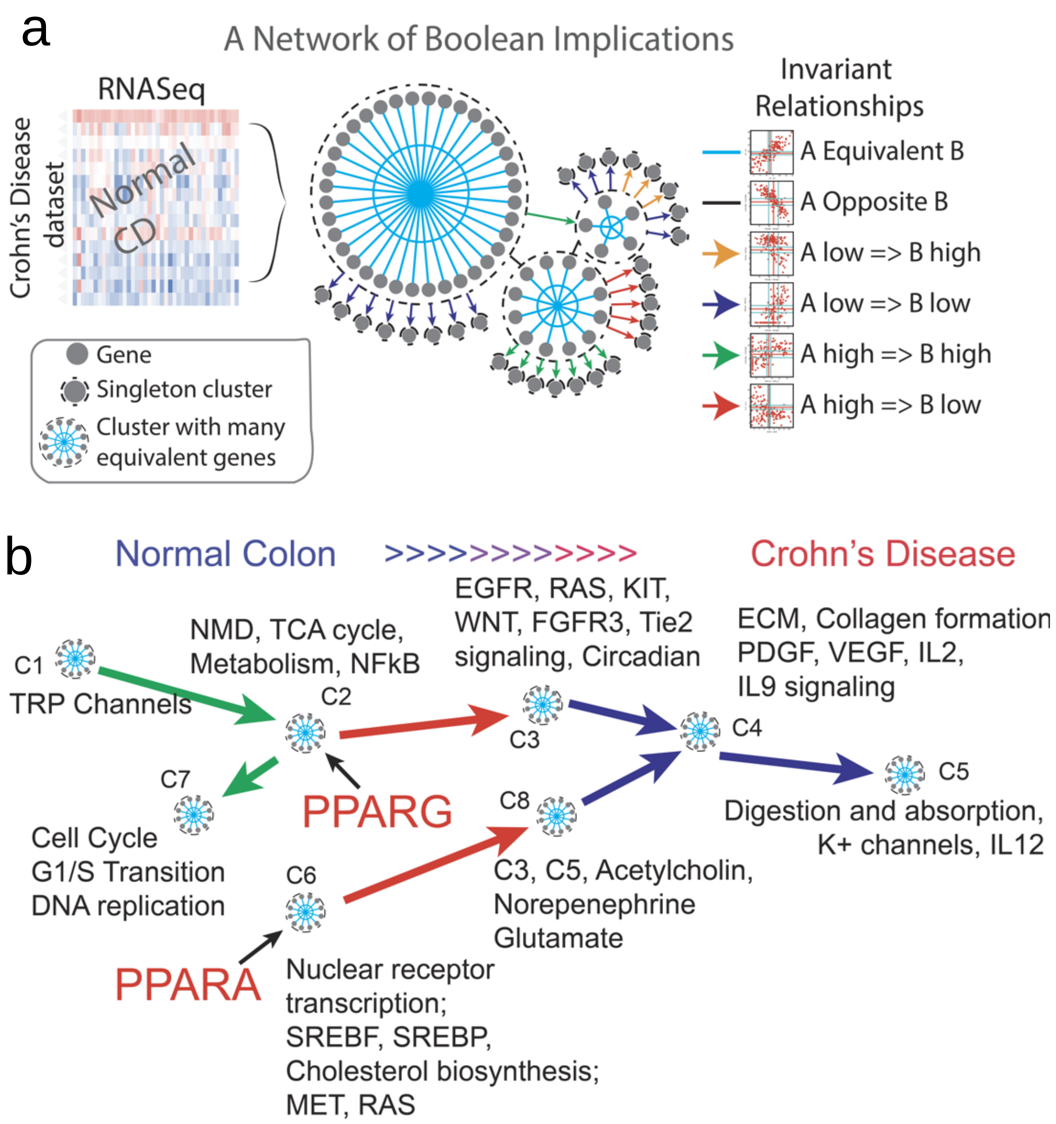

Supplementary Figure 5: Boolean network map of Crohn's disease, CD-map.
(a) Boolean network analysis was performed on Crohn's colitis datasets to identify pathways and gene clusters during CD disease progression. Boolean networks contain six possible Boolean relationships between genes (invariant relationships top right) and genes with similar expression profiles were organized into clusters, and relationships between clusters represented as color-coded edges connecting clusters. **(b)** Schematic of reactome pathway of each cluster of CD-map were performed to understand pathophysiological cellular processes that are enriched during CD progression. *PPARA* is located within cluster #6 and *PPARG* is located within cluster #3. Their stimulation with dual agonists is expected to impact both continuum paths, C2→C3→C4→C5 and C6→C8→C4→C5.

a

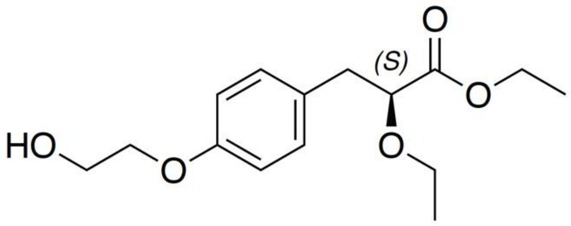

ethyl (S)-2-ethoxy-3-(4-(2-hydroxyethoxy) phenyl)propanoate (compound 1)

b

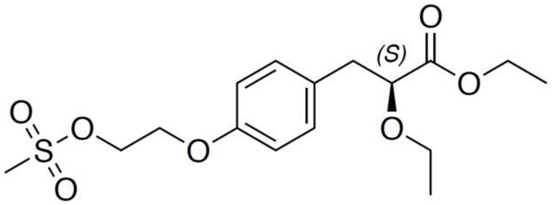

ethyl (S)-2-ethoxy-3-(4-(2-((methylsulfonyl)oxy)ethoxy)phenyl)propanoate (compound 2)

c

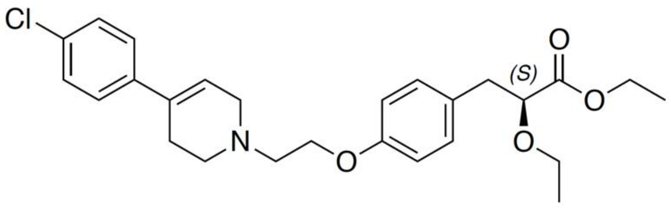

ethyl (S)-3-(4-(2-(4-(4-chlorophenyl)-3,6-dihydropyridin-1(2H)-yl)ethoxy)phenyl)-2-ethoxypropanoate (compound 3)

d

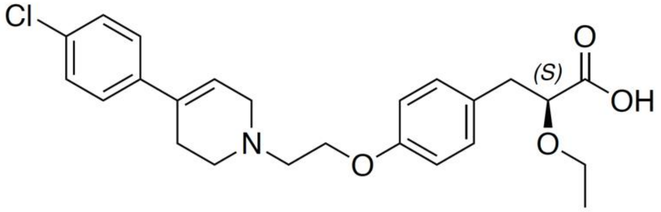

S)-3-(4-(2-(4-(4-chlorophenyl)-3,6-dihydropyridin-1(2H)-yl)ethoxy)phenyl)-2-ethoxypropanoic acid (PAR5359)

Supplementary Figure 6: Synthesis of PAR5359

Structures of intermitant compounds are (a) *ethyl (S)-2-ethoxy-3-(4-(2-hydroxyethoxy) phenyl)propanoate* (compound 1) (b) *ethyl (S)-2-ethoxy-3-(4-(2-((methylsulfonyl)oxy)ethoxy)phenyl)propanoate* (compound 2), (c) *ethyl(S)-3-(4-(2-(4-(4-chlorophenyl)-3,6-dihydropyridin-1(2H)-yl)ethoxy)phenyl)-2-ethoxypropanoate* (compound 3) and (d) final product of PAR5359 *(S)-3-(4-(2-(4-(4-chlorophenyl)-3,6-dihydropyridin-1(2H)-yl)ethoxy)phenyl)-2-ethoxypropanoic acid* (PAR5359).

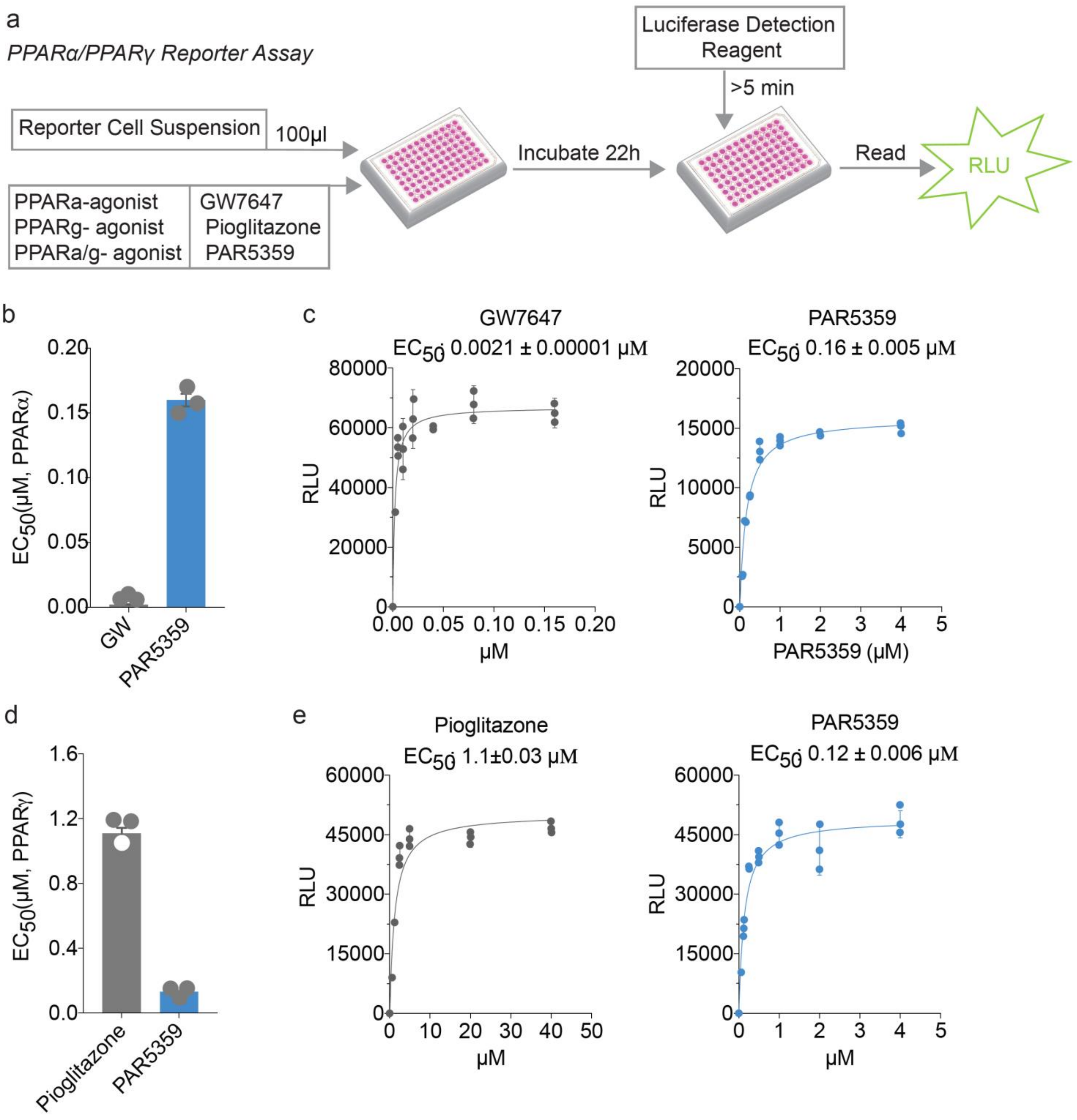

Supplementary Figure 7: **A comparative analysis of potencies of PPARα and PPARγ single and dual agonists.** **(a)** Schematic displaying assay workflow. PPARα and PPARγ reporter cells were dispensed into respective wells of the assay plate and incubated with PPAR agonists (GW7647 and PAR5359 to test PPARα agonist activity and Pioglitazone and PAR5359 to test PPARγ agonist activity). Following 22 h incubation with agonists, treatment media are discarded, and Luciferase Detection Reagent was added (as indicated by manufacture's protocol, Indigo Biosciences). The intensity of light emission (in units of 'Relative Light Units'; RLU) from each assay well was quantified using a plate-reading luminometer. **(b-c)** Bar graph and line graph showing EC_{50} for PPARα and **(d-e)** showing EC_{50} for PPARγ. All results are displayed as mean ± SEM. (n=3).

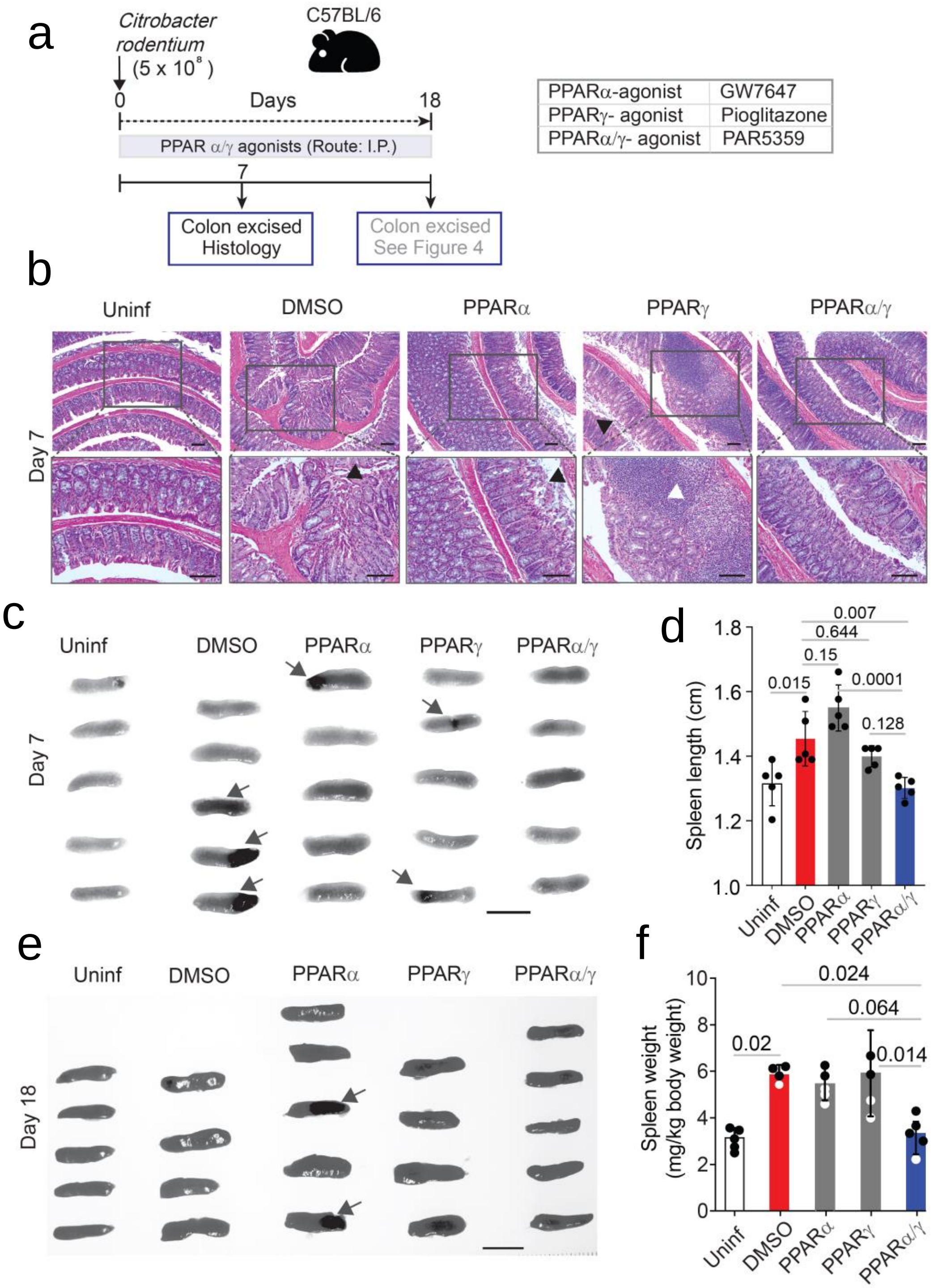

Supplementary Figure 8: **PPARα/γ dual agonists ameliorate *Citrobacter rodentium*-induced infectious colitis in mice (as determined on day #7, i.e., peak inflammation). (a)** Schematic summarizing the workflow for testing PPAR-targeted therapeutics in *C. rodentium*-induced colitis. Mice were gavaged with *C. rodentium* on day 0 and subsequently treated daily with PPAR agonists. Colons were excised on day 7 and analyzed by histology. **(b)** Images display representative fields from H&E-stained colon tissues. Mag = 100x (top) and 200x (bottom), Scale bars 100 μm. White arrowheads point to immune cell infiltrates, whereas black arrowheads point to regions of extensive epithelial destructions and sloughing. **(c-d)** Images in panel c display spleens excised on day 7. Arrows point to black discoloration, likely from splenic infarcts. Scale bar =1 cm. Scatter plots with bar graphs in panel d display the length of the spleens in c. (**e-f**) Images in panel e display spleens excised on day 18. Scale bar =1cm. Arrows point to black discoloration, likely from splenic infarcts. Scatter plots with bar graphs in panel f display the weight of the spleens in e. Statistics: All results are displayed as mean ± SEM. Significance was tested using two-way/one-way ANOVA followed by Tukey's test for multiple comparisons. Significance: *, $p < 0.05$Statistics: All results are displayed as mean ± SEM. Significance was tested using two-way/one-way ANOVA followed by Tukey's test for multiple comparisons. Significance: *, $p < 0.05$; ***, $p < 0.001$. See also Figure 4 for the day 18 results in the *C. rodentium*-induced colitis model

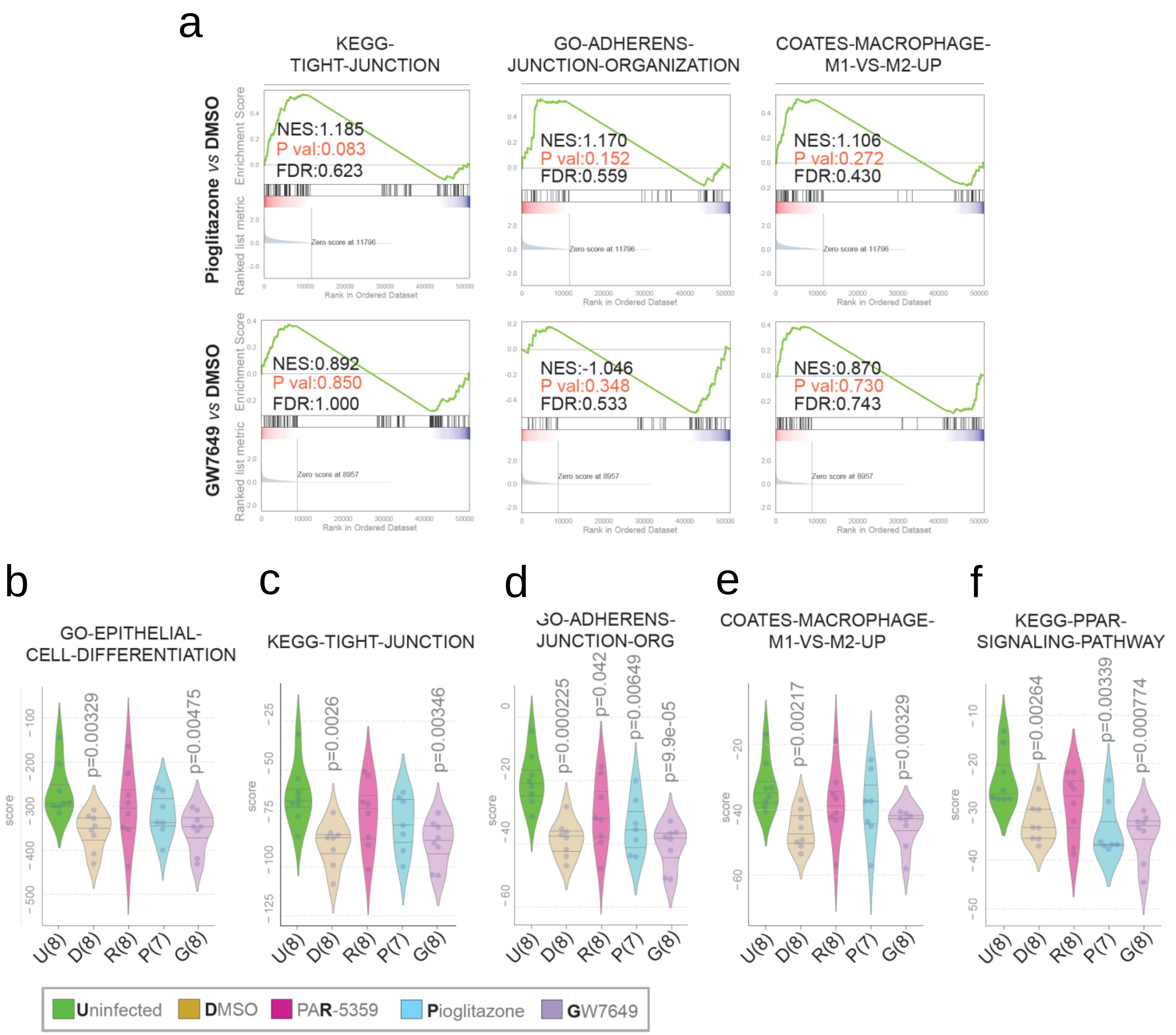

Supplementary Figure 9: **RNA seq analyses of *C. rodentium*-infected colons show that dual agonist PAR5359, but not single agonists GW7647 or Pioglitazone resists *Citrobacter rodentium*-induced gene expression changes.**
(**a**) Pre-ranked GSEA based on pairwise differential expression analyses (Pioglitazone vs DMSO, *top*; GW7647 vs DMSO, *bottom*) are displayed as enrichment plots for epithelial tight (left) and adherens (middle) junction signatures and balanced macrophage processes (right). (**b**-**f**) Violin plots display the deviation of expression of gene sets that represent epithelial differentiation (b), epithelial tight and adherens junctions (c-d), macrophage processes (e) and PPAR signaling (f). In multiple comparisons (b-f), Welch's t-test was used to derive *p* values comparing each condition against the uninfected control condition.

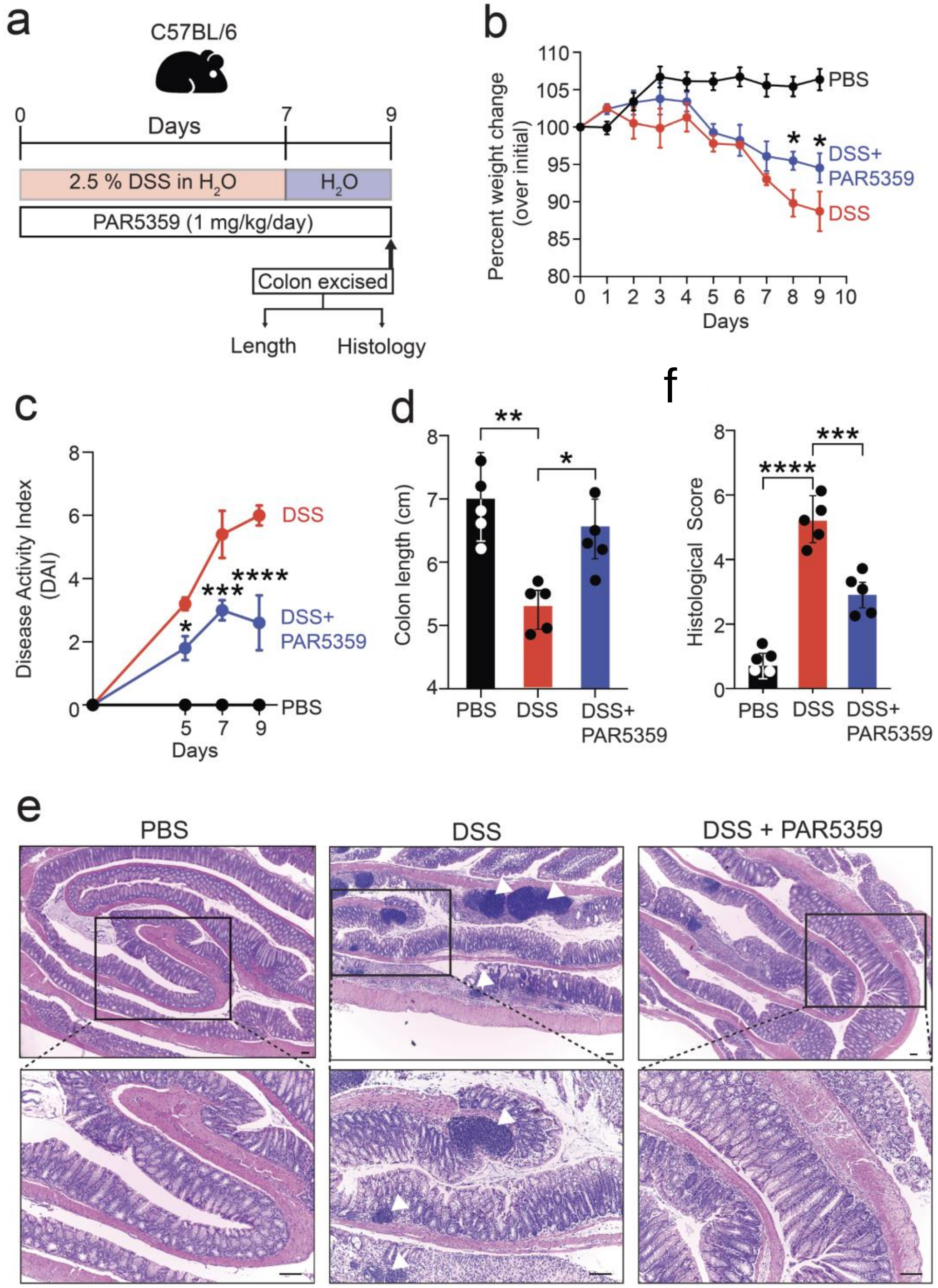

Supplementary Figure 10: PPARα/PPARγ dual agonist, PAR5359 ameliorates DSS-induced colitis in mice. (**a**) Schematic summarizing the workflow for testing PPARα/γ dual agonist (PAR5359) in DSS-induced colitis. Briefly, mice were fed with 2.5% DSS in drinking water for 7 days followed by 2 days of normal drinking water. PAR5359 was given through intrarectal route using an oral gavage needle. The tip of oral gavage needle was greased with medical grade ointment for easy and safe administration. On 9th day all group mice were sacrificed, and colons were analyzed for its morphology, histology and gene expression (RNAseq and qPCR). (**b**) Line graphs display daily weight of mice, from the day of DSS administration (day 1) to the day of sacrifice (day 9). (**c**) Line graphs display disease activity index (DAI) scores, calculated for the days 5, 7 and 9 after DSS administration, which accounts for stool consistency (0-4), rectal bleeding (0-4), and weight loss (0-4). (**d**) Scatter plots with bar graphs display the length of the excised colon at sacrifice (day 9). (**e**) Images representative of H&E-stained sections of the distal colon are shown. Mag = 40x (top) and 100x (bottom); Scale bars, 100 µm . (**f**) Bar graphs showing histological score of H&E sections mentioned in e. White arrowheads = immune cell infiltrates. Statistics: All results are displayed as mean ± SEM. Significance was tested using two-way/one-way ANOVA followed by Tukey's test for multiple comparisons. Significance: *, $p < 0.05$; **, $p < 0.01$, ***, $p < 0.001$, ****, $p < 0.0001$.

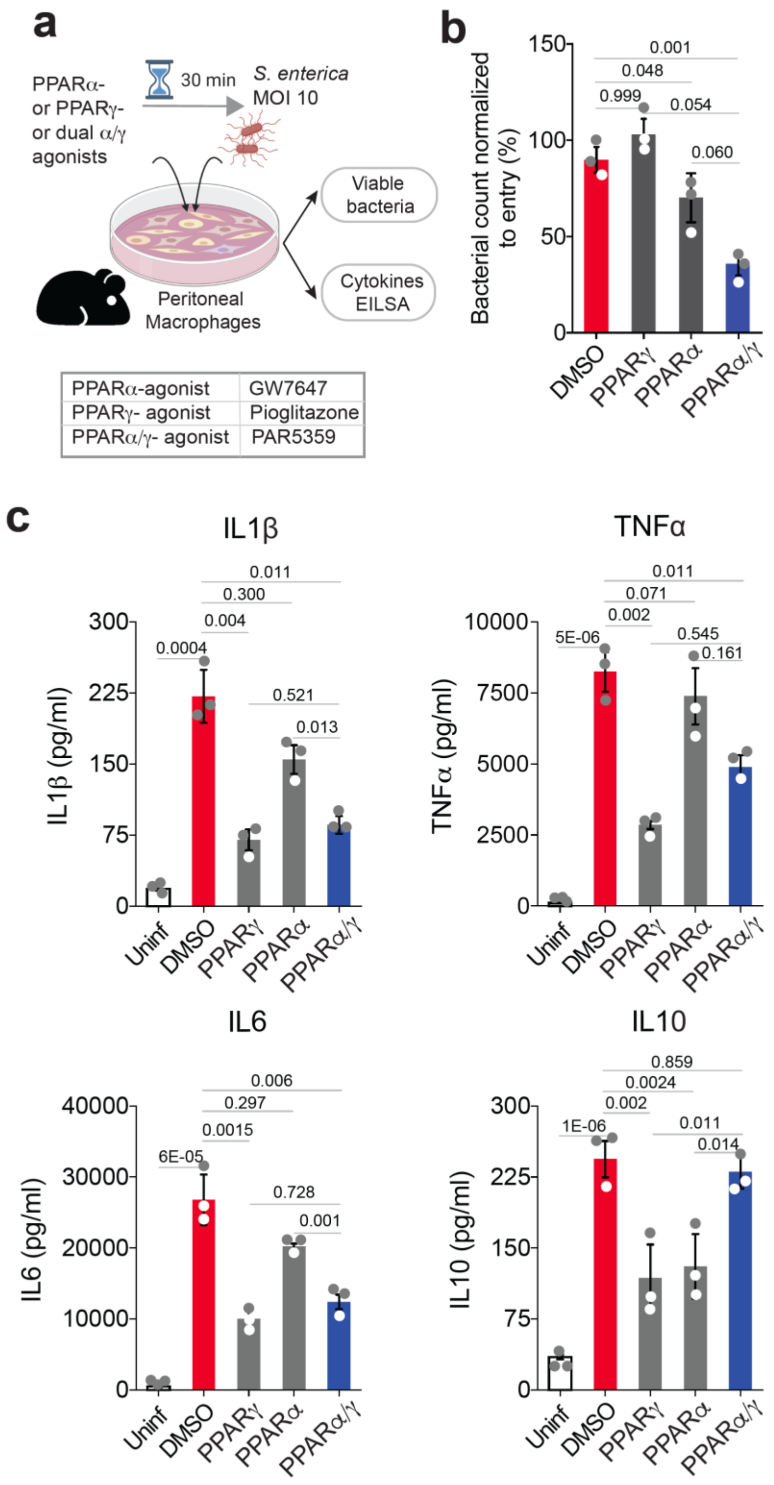

Supplementary Figure 11: PPARα/γ-dual agonists enhance bacterial (*Salmonella enteritica*) clearance. (**a**) Schematic displays the experimental design and workflow. Thioglycolate-induced murine peritoneal macrophages (TG-PM) pretreated with PPAR agonists (see box, below; 20 nM GW7647, 10 µM Pioglitazone and 1 µM PAR5359) were infected with *Salmonella enterica* (*MOI* 10) and subsequently analyzed at 6 h post-infection for bacterial count (Gentamicin protection assay) and secretion of inflammatory cytokines (in supernatant media by ELISA). (**b**) Bar graphs show percent internalized viable bacterial counts of *Salmonella enterica*. (**c**) Bar graphs display the extent of secreted cytokines (IL1β, IL6, TNFα and IL10) in the supernatant media. Statistics: One-way ANOVA followed by Tukey's test for multiple comparisons was performed to test significance. All results are from at least three independent experiments and results displayed as means ± SEM. (n=3), Significance: ns, non-significant, p-value less than 0.05 was considered significant.

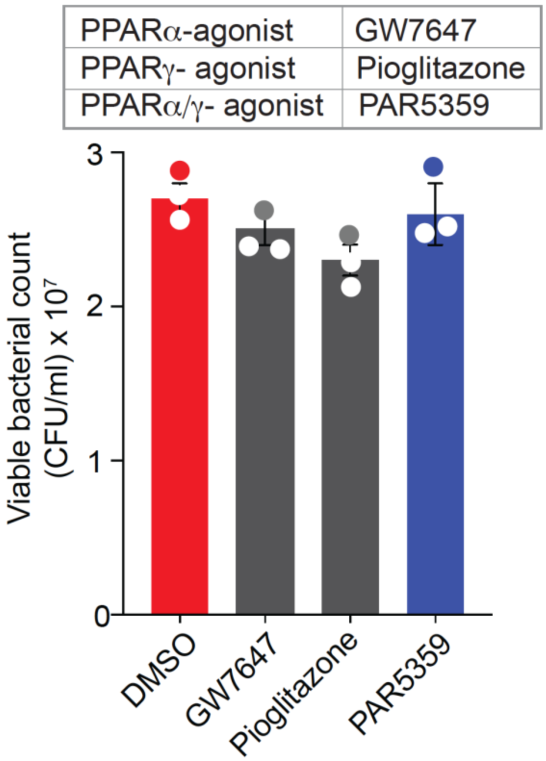

Supplementary Figure 12: PPAR agonists did not affect the viability of AIEC- LF82. *AIEC*-LF82 bacteria (~2 x 10^7) were incubated for 1 h with RPMI media containing 10% FBS and PPAR agonists (see box, below; 20 nM GW7647, 10 mM Pioglitazone and 1 mM PAR5359) at 37°C in CO2 incubator. The culture media was subsequently analyzed for the bacterial count. All results are from three independent experiments and results are displayed as means ± SEM. (n=3). Significance was tested using one-way ANOVA.

TABLES

Supplementary Table 1~~Table S1~~: Table summarizing studies to date and their claims regarding the protective role of PPARγ in IBD

**Interventions/Study models**	**Outcome / Major conclusions**	**Reference**
DSS-induced colitis model in WT and Intestinal endothelial cells (IEC; Villin Cre)-specific PPARγ KO mice<br>Agonist: Rosiglitazone	DSS-induced colitis is worsened in KO mice, compared to WT littermates. PPARγ expressed in the IEC has an endogenous role in protecting against colitis.	(1)
DSS-induced colitis model in WT and CD4+ T cell-specific PPARγ KO mice	DSS-induced colitis is worsened in KO mice, compared to WT littermates. PPARγ in T cells is involved in preventing gut inflammation by regulating adhesion molecules and inflammatory mediators.	(2)
DSS-induced colitis model in WT and Macrophage-specific PPARγ KO mice<br>Agonist: Pioglitazone	Macrophage-specific PPARγ KO exacerbated DSS-induced colitis, impaired Treg compartment, and increased LP CD8+ T cells. In addition, the protective effect of Pioglitazone, the presence of PPARg in macrophages is required.	(3)

Supplementary Table 2~~Table S2~~: Table summarizing studies to date and their conflicting claims regarding the role of PPARα in IBD

Sl. No.	Interventions/Study models	Outcome / Major conclusions	Reference
1.	Dinitrobenzene sulfonic acid (DNBS) induced IBD in WT and PPARα$^{-/-}$ mice.<br>With or without PPARα agonists WY-14643.	DNBS-induced colitis is worsened in KO mice, compared to WT littermates.<br>PPARa and its agonist WY-14643 protects from IBD.<br>**PPARα is protective**	(4)
2.	DSS-induced colitis in Interleukin-10 knockout (IL-10$^{-/-}$)<br>Agonist: Fenofibrate	**PPARα is protective**	(5)
3.	DNBS-induced IBD in WT and PPARα$^{-/-}$ mice, and treatment with dexamethasone, a synthetic glucocorticoid	PPARα enhances the anti-inflammatory effect of dexamethasone.<br>**PPARα is protective**	(6)
4.	DSS-induced colitis in mice treated with<br>Agonist: WY14643	**PPARα is protective**	(7)
6.	DSS-induced colitis in PPARα$^{-/-}$ and WT mice<br>Agonist: WY14643,	PPARα agonist worsens colitis in a PPARα-dependent manner<br>**PPARα is protective**	(8)
7.	DSS and TNBS-induced colitis in WT and PPARα$^{-/-}$mice and *Salmonella typhi* induced colitis, with or without treatment with PPARα agonist.<br>Agonist: Fenofibrate	Increased inflammation in WT, but not KO mice.<br>PPARα agonist worsens colitis in a PPARα-dependent manner<br>**PPARα is harmful**	(9)
9.	DSS-induced colitis in WT and PPARα$^{-/-}$mice; treatment with PPAR agonist<br>Agonist: fenofibrate	Colitis worsened by agonists in WT, but not KO mice.<br>PPARα agonist worsens colitis in a PPARα-dependent manner<br>**PPARα is harmful**	(10)

Supplementary Table 3~~Table S3~~: PPARα/γ dual agonists, their potency and market status.

PPARα/γ Dual Agonist	EC50 (α)	EC50 (γ)	Status
Muraglitazar	320.0nM<br>5680nM	110.0nM<br>243nM	Discontinued (11, 12)
Tesaglitazar	4780nM<br>1200nM	3420nM<br>1300nM	Discontinued (12, 13)
Naveglitazar	2816nM	361nM	Discontinued (14)
Ragaglitazar	3200nM	600nM	Discontinued (15)
Farglitazar	250nM<br>450nM	0.2nM<br>0.34nM	Discontinued (16)
Imiglitazar	8nM	4nM	Discontinued (17)
Netoglitazone	100nM	3000nM	Discontinued (18)
Reglitazar	1900nM	83nM	Discontinued (19)
MK0767	140nM	83nM	Discontinued (20)
KRP-297	850nM	83nM	Discontinued (21)
TZD18	26nM	14nM	Preclinical (22)
Chiglitazar	1200nM	80nM	Phase II clinical trials (23)
Aleglitazar	50nM<br>5nM	21nM<br>9nM	Phase III clinical trials (12)
PLX429	-	-	Preclinical
AVE0847	-	-	Phase II clinical trials
Azaindole-α-alkyloxyphenylpropionic acid	-	-	Preclinical
BVT-142	-	-	Preclinical
O-Arylmandelic acid derivatives	-	-	Preclinical
Amide substituted with α-substituted-β-phenylpropionic acid derivatives	-	-	Preclinical
2-Alkoxydihydro cinnamate derivatives	-	-	Preclinical
LY51029	-	-	Preclinical
α-Aryloxyphenyl acetic acid derivatives	-	-	Preclinical
Tricyclic-α-alkyloxyphenyl propionic acids	-	-	Preclinical
Saroglitazar	0.00065nM	3nM	Phase II clinical trials,<br>Approved in India and Mexico (24)

Supplementary Table 4~~Table S4~~: PPAR agonists and antagonists used in this study.

Common name	Chemical name	structure	EC$_{50}$Potency	Tested in IBD	Reference
**GW7647**<br>PPARα agonist	2-((4-(2-(((Cyclohexylamino)carbonyl)(4-cyclohexylbutyl)amino)ethyl)phenyl)thio)-2-methylpropanoic acid	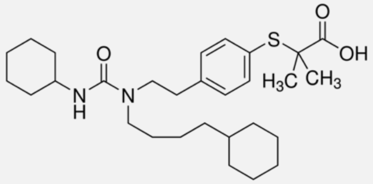	PPARα agonist: 2.1± 0.05 nM	No	(25)<br>(26)
**Pioglitazone**<br>PPARγ agonist	5-(4-(2-(5-ethylpyridin-2-yl)ethoxy)benzyl)thiazolidine-2,4-dione	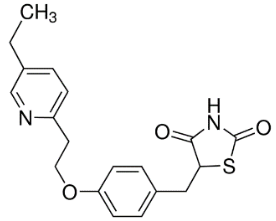	PPARγ agonist: 1.1± 0.03 mM	Yes	(27)
**PAR5359**<br>PPARα/γ dual agonist	3-(4-(2-[4-(4-Chloro-phenyl)-3,6-dihydro-2H-pyridin-1-yl]-ethoxy)-phenyl)-2-ethoxypropionic acid	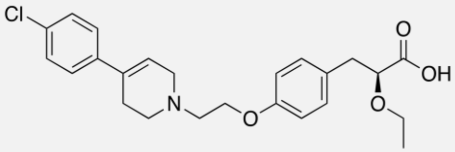	PPARα agonist: 0.16± 0.005 µM<br>PPARγ agonist: 0.12± 0.006 µM	No	(28)
**GW 6471**<br>PPARα antagonist	*N*-((2*S*)-2-(((1*Z*)-1-Methyl-3-oxo-3-(4-(trifluoromethyl)phenyl)prop-1-enyl)amino)-3-(4-(2-(5-methyl-2-phenyl-1,3-oxazol-4-yl)ethoxy)phenyl)propyl)propanamide	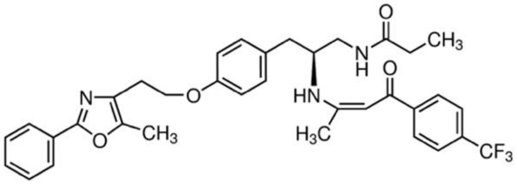	PPARα antagonist: 0.24 mM	No	(29)<br>(30)
**GW 9662**<br>PPARγ antagonist	2-Chloro-5-nitro-N-phenylbenzamide	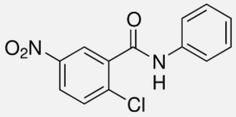	PPARγ antagonist: 3.3 nM	No	(31)

Supplementary Table 5Table S5: Characteristics of IBD patients used as source of PBMCs for use in bacterial clearance assays.

Patient code	Sub-disease	Gender	Treatment history
**CD 76**	Inflammatory	Male	Infliximab current; No prior Biologics
**CD77**	Stricturing	Male	Infliximab in the past
**CD79**	Penetrating	Male	Infliximab in the past
**CD80**	Stricturing	Female	Adalimumab current; No prior Biologics
**CD81**	Penetrating	Female	Infliximab current; Previoulys on Cimzia and Stelara
**CD 82**	Inflammatory	Female	Biologic Naïve
**CD 83**	Stricturing, Penetrating	Male	Infliximab current; No prior Biologics
**UC72**	UC	Female	Biologic Naïve
**UC73**	UC	Female	Biologic Naïve
**UC 74**	UC	Female	Biologic Naïve
**UC 75**	UC	Female	Biologic Naïve
**UC 76**	UC	Female	Off therapy, previous had one dose of Entyvio
**UC 77**	UC	Female	Adalimumab; No prior Biologics

Supplementary Table 6~~Table S6~~: Key resource table

REAGENT or RESOURCE	SOURCE	IDENTIFIER
Biological Samples and Cell Lines		
PPARα reporter assay system	Indigo Biosciences	IB00111-32
PPARγ reporter assay system	Indigo Biosciences	IB00101-32
E. coli LF82 (AIEC-LF82)	Prof. Arlette Darfeuille-Michaud	(32)
Salmonella enteric serovar typhimurium strain SL1344	ATCC	700720
Chemicals and Reagents		
PAR5359	This study (see *Methods*)	
GW7647 (PPARα agonist)	Tocris bioscience	1677
Pioglitazone (PPARγ agonist)	Selleck Chemicals	S2590
GW 6471 (PPARα antagonist)	Tocris bioscience	4618
GW 9662 (PPARγ antagonist)	Tocris bioscience	1508
Lipopolysaccharide (E. coli O111:B4)	Sigma-Aldrich	L4391
PowerUpä SYBR[ä] Green Master Mix	Applied Biosciences	A25741
qScript[ä] cDNA SuperMix	QuantaBio	101414
Direct-zol RNA Miniprep Kit	Zymo Research	R1051
TRIzol[ä] Reagent	Invitrogen	15596018
ELISA MAX[ä] Deluxe Set Mouse IL-6	BioLegend	431304
ELISA MAX[ä] Deluxe Set Mouse IL-1b	BioLegend	432604
ELISA MAX[ä] Deluxe Set Mouse IL-10	BioLegend	431414
ELISA MAX[ä] Deluxe Set Mouse TNF-α	BioLegend	
Dextran Sulfate Sodium Salt (Colitis Grade)	MP Biomedicals, LLC	160110
Hemoccult II	Beckman Coulter	61130
Zinc Formalin Fixative	Sigma-Aldrich	Z2902
ROS Detection Cell-Based Assay Kit (DHE)	Cayman Chemical	601290
Primers		

Species/Targets	Forward primer (5’→ 3’)	Reverse primer (3’→ 5’)
Mouse IL-6 qPCR primers	TGGAGTCACAGAAGGAGTGGCTAAG	TCTGACCACAGTGAGGAATGTCCAC
Mouse IL-1b qPCR primers	GCCTTGGGCCTCAAAGGAAAGAATC	GGAAGACACAGATTCCATGGTGAAG
Mouse TNFa qPCR primers	ATAGCTCCCAGAAAAGCAAGC	CACCCCGAAGTTCAGTAGACA
Mouse IL-10 qPCR primers	CCCTGGGTGAGAAGCTGAAG	CACTGCCTTGCTCTTATTTTCACA
Mouse 18S qPCR primers	GTAACCCGTTGAACCCCATT	CCATCCAATCGGTAGTAGCG
Human PPARA	CATTACGGAGTCCACGCGT	ACCAGCTTGAGTCGAATCGTT
Human PPARG	GAGAAGGAGAAGCTGTTGGC	ATGGCCACCTCTTTGCTCT

Human PPARGC1A	GCTACGAGGAATATCAGCACGA	ACACGGCGCTCTTCAATTG
Software		
Prism	GraphPad	https://www.graphpad.com/scientific-software/prism/
Illustrator	Adobe	https://www.adobe.com/products/illustrator.html
ImageStudio Lite	LI-COR	https://www.licor.com/bio/image-studio-lite/

291

SUPPLEMENTARY REFERNCES

1. Mohapatra SK, Guri AJ, Climent M, Vives C, Carbo A, Horne WT, et al. Immunoregulatory actions of epithelial cell PPAR gamma at the colonic mucosa of mice with experimental inflammatory bowel disease. *PLoS One.* 2010;5(4):e10215.
2. Guri AJ, Mohapatra SK, Horne WT, 2nd, Hontecillas R, and Bassaganya-Riera J. The role of T cell PPAR gamma in mice with experimental inflammatory bowel disease. *BMC Gastroenterol.* 2010;10:60.
3. Hontecillas R, Horne WT, Climent M, Guri AJ, Evans C, Zhang Y, et al. Immunoregulatory mechanisms of macrophage PPAR-gamma in mice with experimental inflammatory bowel disease. *Mucosal Immunol.* 2011;4(3):304-13.
4. Cuzzocrea S, Di Paola R, Mazzon E, Genovese T, Muia C, Centorrino T, et al. Role of endogenous and exogenous ligands for the peroxisome proliferators activated receptors alpha (PPAR-alpha) in the development of inflammatory bowel disease in mice. *Lab Invest.* 2004;84(12):1643-54.
5. Lee JW, Bajwa PJ, Carson MJ, Jeske DR, Cong Y, Elson CO, et al. Fenofibrate represses interleukin-17 and interferon-gamma expression and improves colitis in interleukin-10-deficient mice. *Gastroenterology.* 2007;133(1):108-23.
6. Riccardi L, Mazzon E, Bruscoli S, Esposito E, Crisafulli C, Di Paola R, et al. Peroxisome proliferator-activated receptor-alpha modulates the anti-inflammatory effect of glucocorticoids in a model of inflammatory bowel disease in mice. *Shock.* 2009;31(3):308-16.
7. Azuma YT, Nishiyama K, Matsuo Y, Kuwamura M, Morioka A, Nakajima H, et al. PPARalpha contributes to colonic protection in mice with DSS-induced colitis. *Int Immunopharmacol.* 2010;10(10):1261-7.
8. Zhou X, Cao L, Jiang C, Xie Y, Cheng X, Krausz KW, et al. PPARalpha-UGT axis activation represses intestinal FXR-FGF15 feedback signalling and exacerbates experimental colitis. *Nat Commun.* 2014;5:4573.
9. Qi Y, Jiang C, Tanaka N, Krausz KW, Brocker CN, Fang ZZ, et al. PPARalpha-dependent exacerbation of experimental colitis by the hypolipidemic drug fenofibrate. *Am J Physiol Gastrointest Liver Physiol.* 2014;307(5):G564-73.
10. Gu X, Song Y, Chai Y, Lu F, Gonzalez FJ, Fan G, et al. GC-MS metabolomics on PPARalpha-dependent exacerbation of colitis. *Mol Biosyst.* 2015;11(5):1329-37.
11. Devasthale PV, Chen S, Jeon Y, Qu F, Shao C, Wang W, et al. Design and synthesis of N-[(4-methoxyphenoxy)carbonyl]-N-[[4-[2-(5- methyl-2-phenyl-4-oxazolyl)ethoxy]phenyl]methyl]glycine [Muraglitazar/BMS-298585], a novel peroxisome proliferator-activated receptor alpha/gamma dual agonist with efficacious glucose and lipid-lowering activities. *J Med Chem.* 2005;48(6):2248-50.
12. Dietz M, Mohr P, Kuhn B, Maerki HP, Hartman P, Ruf A, et al. Comparative molecular profiling of the PPARalpha/gamma activator aleglitazar: PPAR selectivity, activity and interaction with cofactors. *ChemMedChem.* 2012;7(6):1101-11.
13. Information. NCfB. Tesaglitazar, CID=208901. https://pubchem.ncbi.nlm.nih.gov/compound/Tesaglitazar. Accessed July 22, 2021.
14. Information. NCfB. Naveglitazar, CID=9888484. https://pubchem.ncbi.nlm.nih.gov/compound/Naveglitazar. Accessed July 22, 2021.
15. Information. NCfB. Ragaglitazar, CID=447458. https://pubchem.ncbi.nlm.nih.gov/compound/Ragaglitazar Accessed July 22, 2021.

16. Nevin DK, Lloyd DG, and Fayne D. Rational targeting of peroxisome proliferating activated receptor subtypes. *Curr Med Chem.* 2011;18(36):5598-623.

17. Information NCfB. Imiglitazar, CID=9890879. https://pubchem.ncbi.nlm.nih.gov/compound/Imiglitazar. Accessed July 22, 2021.

18. Lazarenko OP, Rzonca SO, Suva LJ, and Lecka-Czernik B. Netoglitazone is a PPAR-gamma ligand with selective effects on bone and fat. *Bone.* 2006;38(1):74-84.

19. Information. NCfB. Reglitazar, CID=154000. https://pubchem.ncbi.nlm.nih.gov/compound/Reglitazar. Accessed July 22, 2021.

20. Willson TM, Brown PJ, Sternbach DD, and Henke BR. The PPARs: from orphan receptors to drug discovery. *J Med Chem.* 2000;43(4):527-50.

21. Information NCfB. KRP-297, CID=151183. https://pubchem.ncbi.nlm.nih.gov/compound/krp-297. Accessed July 22, 2021.

22. Information. NCfB. TZD18, CID=9827261. https://pubchem.ncbi.nlm.nih.gov/compound/tzd18. . Accessed July 22, 2021.

23. Li PP, Shan S, Chen YT, Ning ZQ, Sun SJ, Liu Q, et al. The PPARalpha/gamma dual agonist chiglitazar improves insulin resistance and dyslipidemia in MSG obese rats. *Br J Pharmacol.* 2006;148(5):610-8.

24. Jain MR, Giri SR, Trivedi C, Bhoi B, Rath A, Vanage G, et al. Saroglitazar, a novel PPARα/γ agonist with predominant PPARα activity, shows lipid-lowering and insulin-sensitizing effects in preclinical models. *Pharmacol Res Perspect.* 2015;3(3):e00136.

25. Muoio DM, Way JM, Tanner CJ, Winegar DA, Kliewer SA, Houmard JA, et al. Peroxisome proliferator-activated receptor-alpha regulates fatty acid utilization in primary human skeletal muscle cells. *Diabetes.* 2002;51(4):901-9.

26. Paukkeri EL, Leppänen T, Sareila O, Vuolteenaho K, Kankaanranta H, and Moilanen E. PPARalpha agonists inhibit nitric oxide production by enhancing iNOS degradation in LPS-treated macrophages. *Br J Pharmacol.* 2007;152(7):1081-91.

27. Takagi T, Naito Y, Tomatsuri N, Handa O, Ichikawa H, Yoshida N, et al. Pioglitazone, a PPAR-gamma ligand, provides protection from dextran sulfate sodium-induced colitis in mice in association with inhibition of the NF-kappaB-cytokine cascade. *Redox Rep.* 2002;7(5):283-9.

28. Kim MK, Chae YN, Son MH, Kim SH, Kim JK, Moon HS, et al. PAR-5359, a well-balanced PPARalpha/gamma dual agonist, exhibits equivalent antidiabetic and hypolipidemic activities in vitro and in vivo. *Eur J Pharmacol.* 2008;595(1-3):119-25.

29. Redlich S, Ribes S, Schütze S, and Nau R. Palmitoylethanolamide stimulates phagocytosis of Escherichia coli K1 by macrophages and increases the resistance of mice against infections. *J Neuroinflammation.* 2014;11:108.

30. Crane DD, Ireland R, Alinger JB, Small P, and Bosio CM. Lipids derived from virulent Francisella tularensis broadly inhibit pulmonary inflammation via toll-like receptor 2 and peroxisome proliferator-activated receptor α. *Clin Vaccine Immunol.* 2013;20(10):1531-40.

31. He X, Liu W, Shi M, Yang Z, Zhang X, and Gong P. Docosahexaenoic acid attenuates LPS-stimulated inflammatory response by regulating the PPARγ/NF-κB pathways in primary bovine mammary epithelial cells. *Res Vet Sci.* 2017;112:7-12.

32. Darfeuille-Michaud A, Boudeau J, Bulois P, Neut C, Glasser AL, Barnich N, et al. High prevalence of adherent-invasive Escherichia coli associated with ileal mucosa in Crohn's disease. *Gastroenterology.* 2004;127(2):412-21.
