# Supplementary material for: Artificial intelligence-rationalized balanced PPARα/γ dual agonism resets dysregulated macrophage processes in inflammatory bowel disease
Source: Commun Biol. 2022 Mar 14;5:231. doi: 10.1038/s42003-022-03168-4 (PMC8921270; doi:10.1038/s42003-022-03168-4)
Supplement: Supplementary file 3 — Description of Additional Supplementary Files [file 42003_2022_3168_MOESM3_ESM.pdf]

Description of Additional Supplementary Files

File name: Supplementary Data 1
Description: Source data underlying the graphs and charts presented in the main figures.
