# Supplementary material for: Artificial intelligence-rationalized balanced PPARα/γ dual agonism resets dysregulated macrophage processes in inflammatory bowel disease
Source: Commun Biol. 2022 Mar 14;5:231. doi: 10.1038/s42003-022-03168-4 (PMC8921270; doi:10.1038/s42003-022-03168-4)
Supplement: Supplementary file 5 — Reporting Summary [file 42003_2022_3168_MOESM5_ESM.pdf]

nature portfolio

Corresponding author(s): William Sandborn
Soumita Das
Debashis Sahoo
Pradipta Ghosh

Last updated by author(s): 2022/02/03

Reporting Summary

Nature Portfolio wishes to improve the reproducibility of the work that we publish. This form provides structure for consistency and transparency in reporting. For further information on Nature Portfolio policies, see our Editorial Policies and the Editorial Policy Checklist.

Statistics

For all statistical analyses, confirm that the following items are present in the figure legend, table legend, main text, or Methods section.

n/a	Confirmed	
☐	☒	The exact sample size (*n*) for each experimental group/condition, given as a discrete number and unit of measurement
☐	☒	A statement on whether measurements were taken from distinct samples or whether the same sample was measured repeatedly
☐	☒	The statistical test(s) used AND whether they are one- or two-sided<br>*Only common tests should be described solely by name; describe more complex techniques in the Methods section.*
☐	☒	A description of all covariates tested
☐	☒	A description of any assumptions or corrections, such as tests of normality and adjustment for multiple comparisons
☐	☒	A full description of the statistical parameters including central tendency (e.g. means) or other basic estimates (e.g. regression coefficient) AND variation (e.g. standard deviation) or associated estimates of uncertainty (e.g. confidence intervals)
☐	☒	For null hypothesis testing, the test statistic (e.g. *F*, *t*, *r*) with confidence intervals, effect sizes, degrees of freedom and *P* value noted<br>*Give P values as exact values whenever suitable.*
☒	☐	For Bayesian analysis, information on the choice of priors and Markov chain Monte Carlo settings
☒	☐	For hierarchical and complex designs, identification of the appropriate level for tests and full reporting of outcomes
☒	☐	Estimates of effect sizes (e.g. Cohen's *d*, Pearson's *r*), indicating how they were calculated

Our web collection on statistics for biologists contains articles on many of the points above.

Software and code

Policy information about availability of computer code

Data collection	All datasets are publicly available in Gene Expression Omnibus.
Data analysis	Instructions for how to analyze dataset is available at https://github.com/sahoo00/BoNE

For manuscripts utilizing custom algorithms or software that are central to the research but not yet described in published literature, software must be made available to editors and reviewers. We strongly encourage code deposition in a community repository (e.g. GitHub). See the Nature Portfolio guidelines for submitting code & software for further information.

Data

Policy information about availability of data

All manuscripts must include a data availability statement. This statement should provide the following information, where applicable:

- Accession codes, unique identifiers, or web links for publicly available datasets
- A description of any restrictions on data availability
- For clinical datasets or third party data, please ensure that the statement adheres to our policy

Data Availability
The codes are publicly available at the following links: https://github.com/sahoo00/BoNE; https://github.com/sahoo00/Hegemon. The data supporting the findings of this study are available within the paper is available in Supplementary Data 1. The GEO datasets will be embargoed for one year after publication and released to readers upon request to the corresponding authors. Publicly available datasets used: GSE83687, GSE73661, GSE16879, GSE59071, GSE48958, GSE50594, GSE37283, E-MTAB-7604, GSE42768, E-MTAB-5249, GSE53835, GSE90577, GSE87317, GSE27302, GSE39859, GSE107933, GSE65408, GSE73661, GSE16879, GSE59071, GSE48958, GSE59071, GSE48958, GSE73661, GSE16879, GSE50594, E-MTAB-7604, GSE37283, GSE134312, GSE63626, GSE24759, GSE31255, GSE24759, GSE134312, GSE63626, GSE119087.

Field-specific reporting

Please select the one below that is the best fit for your research. If you are not sure, read the appropriate sections before making your selection.

- [x] Life sciences
- [ ] Behavioural & social sciences
- [ ] Ecological, evolutionary & environmental sciences

For a reference copy of the document with all sections, see nature.com/documents/nr-reporting-summary-flat.pdf

Life sciences study design

All studies must disclose on these points even when the disclosure is negative.

Sample size	All available samples were used.
Data exclusions	No data were excluded from the analysis.
Replication	At least three biological replicates were used in each experiments.
Randomization	Sample allocation were controlled based on disease subtype. Healthy, UC and CD samples were used.
Blinding	Blinding was not possible. Investigator knew the disease status of each samples.

Reporting for specific materials, systems and methods

We require information from authors about some types of materials, experimental systems and methods used in many studies. Here, indicate whether each material, system or method listed is relevant to your study. If you are not sure if a list item applies to your research, read the appropriate section before selecting a response.

Materials & experimental systems

n/a	Involved in the study	
[ ]	[x]	Antibodies
[ ]	[x]	Eukaryotic cell lines
[x]	[ ]	Palaeontology and archaeology
[ ]	[x]	Animals and other organisms
[ ]	[x]	Human research participants
[x]	[ ]	Clinical data
[x]	[ ]	Dual use research of concern

Methods

n/a	Involved in the study	
[x]	[ ]	ChIP-seq
[x]	[ ]	Flow cytometry
[x]	[ ]	MRI-based neuroimaging

Antibodies

Antibodies used	ELISA MAXä Deluxe Set Mouse IL-6 BioLegend 431304<br>ELISA MAXä Deluxe Set Mouse IL-1b BioLegend 432604<br>ELISA MAXä Deluxe Set Mouse IL-10 BioLegend 431414<br>ELISA MAXä Deluxe Set Mouse TNF- BioLegend 430904
Validation	To apply Validation done before see the use.

ELISA MAXä Deluxe Set Mouse IL-6 BioLegend 431304
ELISA MAXä Deluxe Set Mouse IL-1b BioLegend 432604
ELISA MAXä Deluxe Set Mouse IL-10 BioLegend 431414
ELISA MAXä Deluxe Set Mouse TNF- BioLegend 430904

Eukaryotic cell lines

Policy information about cell lines

Cell line source(s)	RAW 264.7 cells
Authentication	NA
Mycoplasma contamination	Regular test for mycobacteriulm has bee done to check it contamination.
Commonly misidentified lines (See ICLAC register)	*Name any commonly misidentified cell lines used in the study and provide a rationale for their use.*

Animals and other organisms

Policy information about studies involving animals; ARRIVE guidelines recommended for reporting animal research

Laboratory animals	Mice
Wild animals	This study did not involve wild animals.
Field-collected samples	This study did not involve samples collected from the field.
Ethics oversight	Animals were bred, housed, and euthanized according to University of California San Diego Institutional Animal Care and Use Committee (IACUC) policies and guidelines.

Note that full information on the approval of the study protocol must also be provided in the manuscript.

Human research participants

Policy information about studies involving human research participants

Population characteristics	Ulcerative colitis and Chron's disease patients:<br>Age : 27-71 years.<br>Gender: both male and female.<br>Ethnicity: Caucasian, African American and Middle Eastern.<br>Disease location: Ileocolitis, colitis, and Ileitis including stricturing, non-stricturing, non-penetrating, and penetrating.<br>Disease Duration: 1-23 years.<br>Drug history: case dependent, either naive or include the use of Remicade, Humira, Adalimumab, Infliximab, and Vedolizumab. Some cases have past history of Adalimumab, Infliximab, and Vedolizumab.
Recruitment	Blood sample collection: For the recruitment, subjects are identified by the treating physician who has a deep understanding of the pathophysiology and clinical practice guidelines when it comes to the GI conditions. The process of identification will involve checking the patient's chart for study eligibility, mainly why the procedure was scheduled by the patient's physician to judge if the patient meet's inclusion criteria, and whether existing co-morbidities warrant exclusion from the study. We have used the following inclusion and exclusion criteria.<br>Inclusion Criteria: Consented to the study; Healthy or diseased, where tissue can be endoscopically accessed; Patients presenting with routine requests for diagnostic or screening endoscopic procedures; any gender, ethnicity and socio-economic background.<br>Exclusion Criteria: Impaired decisional capacity, comprehension, cognition; known bleeding diathesis, such as von Willebrand's disease or hemophilla; Any other illness that impairs, as a secondary complication platelet function or number, Pregnancy (beta HCG testing is done routinely before endoscopy); Declined consent.<br>Patients are consented at least 2 hours prior to the procedure either in the pre-procedure room or in the procedure room. Moreover, study subjects are enrolled at the time when they present for routine endoscopic procedures (interrogating the upper as well as lower GI tract) after taking the necessary preparation for such a procedure. They have to have undergone prior assessment for fitness to undergo the procedure as per routine procedure guidelines. The procedures should have been ordered during the course of routine standard of care, as determined by their treating physicians, with no influence whatsoever from the endoscopist who will enroll patients into the study and obtain informed consent. In many cases, several healthy individuals undergoing routine screening procedures are consented as potential sources for healthy "control/normal" tissues but may be found to have disease conditions. Thus, informed consent includes such disclosure that depending on the endoscopic findings, the nature of participation in the study may change in those cases. The risk is low because the decision to get the procedure done and the indication is determined by another physician/team other than the PI who is conducting this study/obtaining informed consent. However, to further minimize risk, it is clarified that the decision to participate in the study is entirely voluntary and can not affect management/care decisions. i.e. the study participant can not get any different care if they choose not to participate.
Ethics oversight	University of California San Diego, Human Research Protection Program (HRPP) Institutional Review Board Project no 160246

Note that full information on the approval of the study protocol must also be provided in the manuscript.
